# Preoperative voice analysis and survival outcomes in papillary thyroid cancer with recurrent laryngeal nerve invasion

**DOI:** 10.3389/fendo.2022.1041538

**Published:** 2022-10-27

**Authors:** Jiaming Chen, Qi Zhong, Lizhen Hou, Hongzhi Ma, Qian Shi, Ling Feng, Shizhi He, Yansong Lin, Meng Lian, Xixi Shen, Ru Wang, Jugao Fang

**Affiliations:** ^1^ Department of Otorhinolaryngology Head and Neck Surgery, Beijing Tongren Hospital, Capital Medical University, Beijing, China; ^2^ Department of Nuclear Medicine, Peking Union Medical College Hospital, Peking Union Medical College, Chinese Academy of Medical Sciences, Beijing, China

**Keywords:** acoustic parameters, recurrence, risk factors, recurrent laryngeal nerve, thyroid neoplasms

## Abstract

**Objective:**

To investigate the clinicopathological characteristics of papillary thyroid cancer (PTC) and identify risk factors for postoperative recurrence of PTC with recurrent laryngeal nerve (RLN) involvement.

**Methods:**

In total, 171 patients (112 women and 59 men, age: 18–80 years, and 65 patients aged ≥ 55) with T4a PTC with RLN involvement, treated at Beijing Tongren Hospital, Capital Medical University, from January 2006 to December 2020, were retrospectively examined. Clinicopathological characteristics, including voice analysis results, and survival outcomes were assessed. The Mann–Whitney U and Kruskal–Wallis H tests were used to analyze differences in acoustic parameters. The Kaplan–Meier method was used to calculate the overall survival (OS) and recurrence-free (RFS) rates. Univariate and multivariate Cox regression analyses were performed of the clinical data.

**Results:**

The postoperative follow-up period ranged from 12 to 196 months (mean: 66.18 months). Of the 171 patients, 16 had recurrence and 8 died of thyroid-related diseases. The 5-year OS rate was 95.22%. The 5-year RFS rate was 89.38%. Jitter and shimmer were higher and maximum phonation time was shorter in patients with preoperative vocal cord paralysis (VCP) than in those without RLN involvement, and in those with RLN involvement but without preoperative VCP. Acoustic parameters were similar in patients with no preoperative VCP and those without RLN involvement. Voice analysis results did not differ between cases with RLN adhesion and RLN invasion. Univariate analysis showed that age at onset ≥ 55 years, preoperative RLN palsy, and esophageal invasion were risk factors for postoperative recurrence of PTC with RLN involvement. Multivariate analysis showed that onset age ≥ 55 years (OR 4.52, 95% confidence interval: 1.44–14.19, P = 0.010) was an independent risk factor for recurrence.

**Conclusions:**

PTC patients with RLN invasion can achieve good outcomes. Preoperative voice analysis may offer insights into RLN function. Age of onset ≥ 55 years is an independent risk factor for postoperative recurrence in T4a PTC patients.

## Introduction

Thyroid carcinoma is the most common malignant endocrine tumor. In recent years, the morbidity rate of thyroid cancer has gradually increased worldwide. In China, according to the statistics of the Chinese National Cancer Center, the number of newly diagnosed thyroid cancers is estimated to be 220,000 per year, causing it to rank seventh among all malignant tumors (1). Although the incidence of thyroid cancer is high, the associated mortality rate is rather low, mainly because most thyroid cancers are papillary thyroid cancers (PTC), which often have good differentiation and are associated with a better prognosis.

In clinical practice, some PTCs show high invasiveness, spreading to the recurrent laryngeal nerve (RLN) most commonly. It has been reported that, in PTC patients with extrathyroidal invasion, the rate of RLN involvement is 33–61% (2–4). The involvement of RLN not only affects the survival outcomes of patients with PTC, but it also affects their quality of life. Therefore, surgical management of these patients, to resect the tumor while preserving neural functions, has long been a goal. However, identification of RLN involvement using conventional imaging examinations is difficult.

In this study, we retrospectively evaluated PTC patients with RLN invasion and analyzed their clinicopathological characteristics, preoperative acoustic parameters, and survival outcomes, to identify risk factors for postoperative recurrence of PTC with RLN involvement.

## Methods

### Study populations

This study was approved by the ethics committee of Beijing Tongren Hospital, Capital Medical University.

**Figure 1:**
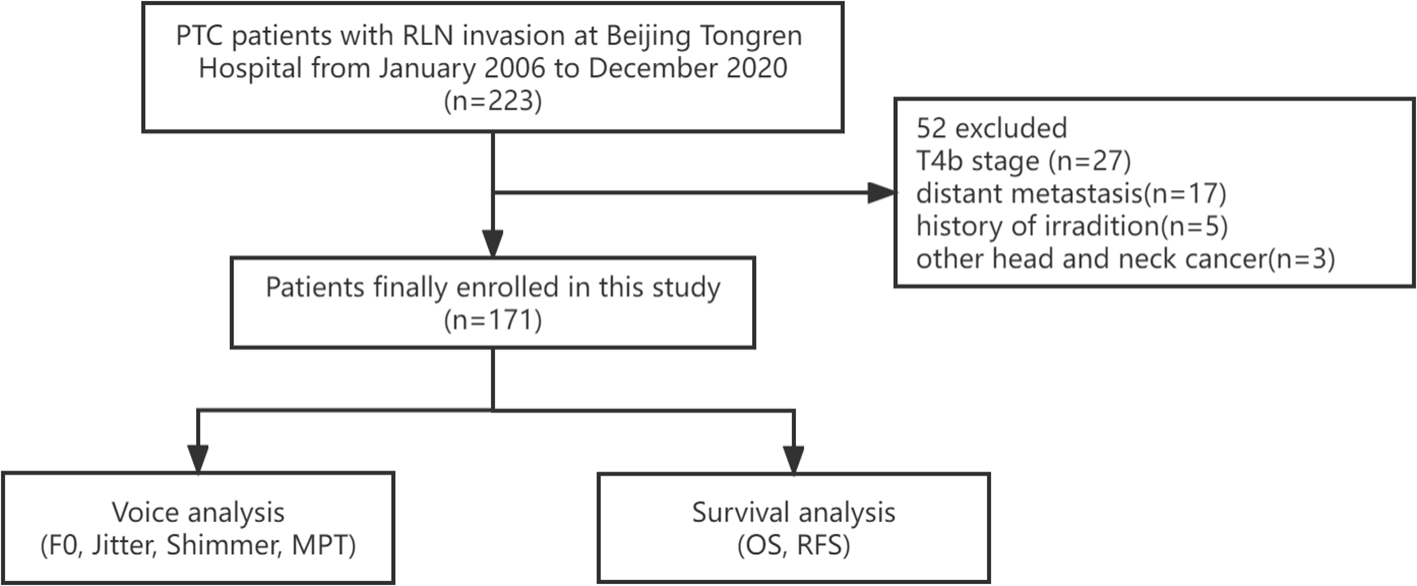
The workflow of this study.

This retrospective study included PTC patients with RLN invasion at Beijing Tongren Hospital from January 2006 to December 2020. The exclusion criteria were as follows: history of irradiation or of other head and neck cancers; T4b patients according to the American Joint Committee on Cancer’s (AJCC) Staging Manual, 8th edition (5); and distant metastasis. The inclusion flow chart of this study is shown in **Figure 1**.

In all, 171 patients with T4a PTC with RLN invasion were enrolled in the analysis: stage I, 106 cases (T4aN0M0: 22 cases, T4aN1aM0: 27 cases, T4aN1bM0: 57 cases); stage III, 65 cases (T4aN0M0: 26 cases, T4aN1aM0: 16 cases, T4aN1bM0: 23 cases). Additionally, 116 PTC patients with stage T3 were enrolled as a control group for voice analysis.

### Preoperative assessment

All patients underwent preoperative high-resolution ultrasound (US) evaluation of thyroid nodules and potential metastatic lymph nodes (LNs) in the neck. When the tumor size was > 4 cm or when there were suspicious metastatic LNs and potential extrathyroidal lesions, computed tomography (CT) or magnetic resonance imaging (MRI) was also performed. A laryngoscopic examination was performed to evaluate the RLN function of each patient.

Some patients also underwent preoperative voice assessment using the multi-dimensional voice program (Version 3.3.0, Key Pentax, Tokyo, Japan). The acoustic parameters included fundamental frequency (F0), percentage of jitter (%), percentage of shimmer (%), and maximum phonation time (MPT). The voice of each patient was recorded three times, and the average of each parameter was used for further analysis.

### Surgical and adjuvant treatment

According to the 2015 American Thyroid Association (ATA) risk scoring system (6), all patients in this study were assigned to the high-risk group, and total thyroidectomy was recommended. For some patients who strongly refused total thyroidectomy, and whose RLNs were mildly invaded, hemithyroidectomy was performed. For LN dissection, prophylactic central neck LN dissection was performed routinely, and lateral neck LN dissection was only performed in patients with high suspicion of lateral LN metastasis on CT and MRI or with biopsy-confirmed nodal metastases.

Preoperative RLN function assessment and intraoperative observation are vital for the treatment of invaded RLNs. When the tumors mildly invaded or adhered to the nerve, the tumor tissue was meticulously peeled off the nerve under magnification using surgical loupes. However, when there was vocal cord paralysis (VCP) preoperatively, or if there was extensive infiltration, the nerve was usually sacrificed (7, 8). Intraoperative nerve monitoring (IONM) was performed in this group. In addition, it should be noted that when bilateral RLN injury was confirmed, tracheotomy was usually performed intraoperatively.

Based on the pathological results, patients underwent radioactive iodine (RAI) with 150–200 mCi at 4–8 weeks after surgery.

### Statistical analyses

Continuous variables were expressed as median and range or mean ± standard deviation, and categorical variables were expressed as numbers and percentages. The Mann–Whitney U test and Kruskal–Wallis H test were used to determine the differences in acoustic parameters between groups. To evaluate the associations between clinicopathological characteristics and recurrence, the χ^2^ test or Fisher’s exact test was utilized. Univariate and multivariate Cox proportional hazards models were also used to determine the risk factors for recurrence. Variables with *P* < 0.1 in the univariate analysis were subsequently included in the multivariable model. The Kaplan–Meier method was used to estimate overall survival (OS) and recurrence-free survival (RFS), and differences were analyzed using the log-rank test. Statistical differences were defined as *P* < 0.050.

## Results

### Patients’ baseline characteristics

From January 2006 to December 2020, 2,782 PTC patients underwent surgery at Beijing Tongren Hospital. Among them, 171 (6.15%) were confirmed to have RLN invasion. There were 112 females and 59 males, aged between 18 and 80 years. Twelve patients had PTC size > 4 cm. Eighty-five patients presented with multifocal lesions. Preoperative laryngoscopy revealed VCP in 60 patients. Thirty-five patients had laryngeal or tracheal invasion, while 29 had esophageal involvement.

When we investigated the RLN invasion condition more closely, we found that the RLNs of 33 patients were invaded by metastatic LNs. RLN invasion occurred in the recurrent laryngeal nerve inlet zone in 22 patients. In 82 patients, the RLN adhered to the tumor, while the RLNs of the other 89 patients were directly invaded by the tumor.

A total of 150 patients underwent total thyroidectomy and 85 patients underwent lateral neck LN dissection. Intraoperative tracheotomy was performed in 16 patients, and among them, 3 patients had bilateral RLN invasion. Finally, 14 patients had their tracheostomy tubes removed, and the average time to removing the tubes was 149.79 ± 102.97 days. A total of 4 patients were treated with endoscopic laser-assisted medial arytenoidectomy. Of the patients that had the RLN spared surgically, 73 patients were treated with separation or partial layer resection. Among these patients, seven patients suffered from transient VCP one week after surgery, while no patients developed permanent vocal cord paralysis one year after surgery. The remaining 98 patients underwent resection of the RLN. Immediate RLN reconstructions were performed in 33 patients, of which 13 patients had direct RLN anastomosis and 21 patients underwent ansa cervicalis nerve-to-RLN anastomosis. At one year after surgery, we found that the 33 patients who received immediate RLN reconstructions recovered their RLN function with varying degrees, while the other 65 patients who did not have RLN reconstructions all presented with VCP.

Pathological results indicated that seven patients had tall cell variant PTC and one patient had follicular variant PTC, while the remaining 163 patients had classic variant PTC. All patients had negative resection margins. A total of 125 patients had LN metastasis. Of these, 33 patients had five or more central metastatic LNs and 80 had lateral metastatic LNs. Seventeen patients were diagnosed with Hashimoto thyroiditis. In total, 126 patients underwent postoperative RAI therapy.

### Survival outcomes

**Figure 2:**
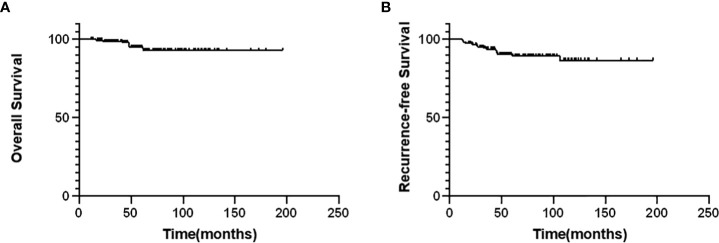
Kaplan–Meier curves for the overall survival **(A)** and recurrence-free survival **(B)**.

During follow-up, recurrence was observed in 16 of 171 patients (9.36%). Among these, recurrence involved the thyroid lobe and the surgical bed in three patients; larynx, trachea, or esophagus in five patients; and neck LNs in eight patients. Nine patients underwent a second operation, and one patient received targeted therapy. Eight patients died of thyroid-related disease. The average follow-up was 66.18 months, with a median of 58 months. As shown in **Figure 2**, the 5-year OS rate was 95.22% and the 5-year RFS rate was 89.38%.

### Acoustic parameters

The differences in acoustic parameters between PTC patients with different RLN conditions are presented in **Tables 1** and **2**. The results showed that patients with preoperative VCP had higher jitter, shimmer, and shorter MPT than patients without RLN involvement, as well as patients with RLN involvement but without preoperative VCP. Patients with RLN involvement and those without preoperative VCP showed no differences in acoustic parameters as compared to patients without RLN involvement.

**Table 1:** The acoustic parameters of PTC patients.

Parameters	RLN condition			H value	*P* value
	Group 1 RLN invasion without preoperative VCP (n = 70)	Group 2 RLN invasion with preoperative VCP (n = 46)	Group 3 Without RLN invasion (n = 116)		
F0 [Hz, *P*50 (*P*25, *P*75)]					
Male	132.53 (121.12, 140.78)	137.24 (121.12, 159.66)	133.72 (118.66, 163.05)	1.83	0.400
Female	215.85 (208.43, 231.54)	221.48 (209.95, 238.42)	223.61 (204.82, 232.24)	7.42	0.025
Jitter [%, *P*50 (*P*25, *P*75)]	1.89 (1.01, 2.69)	2.25 (1.33, 3.19)	1.71 (0.67, 2.58)	37.24	< 0.001
Shimmer [%, *P*50 (*P*25, *P*75)]	5.26 (4.11, 7.27)	5.77 (4.46, 7.71)	5.46 (4.15, 6.68)	11.62	< 0.001
MPT[s, *P*50 (*P*25,*P*75)]	12.00 (10.00, 15.00)	10.00 (8.00, 15.00)	12.00 (10.00, 15.00)	20.93	< 0.001

PTC, papillary thyroid cancer; RLN, recurrent laryngeal nerve; VCP, vocal cord paralysis; MPT, maximum phonation time.

**Table 2:** Pairwise comparison with Bonferroni adjustment.

	Jitter	Shimmer	MPT	F0 (male)	F0 (Female)
Group1 VS Group 2	< 0.001	0.018	< 0.001	–	0.029
Group1 VS Group 3	0.598	1.000	1.000	–	1.000
Group2 VS Group 3	< 0.001	0.003	< 0.001	–	0.038

Group 1, RLN invasion without preoperative VCP; Group 2, RLN invasion with preoperative VCP; Group 3, Without RLN invasion; MPT, maximum phonation time.

In addition, the F0 of female patients with VCP was significantly higher than that of those without RLN involvement (*P* = 0.029) and those without VCP (*P* = 0.038), whereas there was no evident difference in males.

When the severity of RLN invasion differed among the patients, we also attempted to determine the association between acoustic parameters and the extent of RLN invasion. Thus, patients with direct RLN invasion (n = 57) and simple adhesions (n = 59) were enrolled. **Table 3** presents the results. There were no significant differences between the two groups.

**Table 3:** Acoustic parameters of PTC patients with RLN invasion and adhesion.

Parameters	RLN adhesion (n = 59)	RLN invasion (n = 57)	Z value	*P* value
F0 [Hz, *P*50 (*P*25, *P*75)]				
Male	133.41 (121.12, 146.92)	142.08 (123.74, 172.96)	–1.22	0.221
Female	216.48 (204.47, 237.02)	226.33 (213.16, 243.46)	–1.28	0.202
Jitter [%, *P*50 (*P*25, *P*75)]	2.09 (1.16, 2.91)	2.57 (1.49, 3.90)	–1.93	0.054
Shimmer [%, *P*50 (*P*25, *P*75)]	6.09 (4.45, 7.63)	5.66 (4.47, 8.22)	–0.23	0.819
MPT [s, *P*50 (*P*25,*P*75)]	10.00 (7.00, 15.00)	10.00 (8.00, 15.00)	–0.41	0.682

PTC, papillary thyroid carcinoma; RLN, recurrent laryngeal nerve; MPT, maximum phonation time.

### Association between clinicopathological characteristics and recurrence

Among the investigated clinicopathological characteristics, only age ≥ 55 years (*P* = 0.001) and esophageal invasion (*P* = 0.021) differed significantly in terms of recurrence. Other characteristics (such as sex, multifocal tumors, and tumor size) were not associated with recurrence (**Table 4**).

**Table 4:** Association between clinicopathological characteristics and recurrence in PTC patients with RLN invasion (n, %).

Characteristics	No Recurrence (n = 155)	Recurrence (n = 16)	χ^2^	*P*
Age			10.249	0.001
≥ 55 years	53 (34.19)	12 (75.00)		
<55 years	102 (65.81)	4 (25.00)		
Sex			0.070	0.791
Male	53 (34.19)	6 (37.50)		
Female	102 (65.81)	10 (62.50)		
Tumor Size (cm)			0.000	1.000
≥4	11 (7.10)	1 (6.25)		
<4	144 (92.90)	15 (93.75)		
Multifocal disease			0.001	0.980
Yes	77 (49.68)	8 (50.00)		
No	78 (50.32)	8 (50.00)		
Preoperative VCP			3.471	0.062
Yes	51 (32.90)	9 (56.25)		
No	104 (67.10)	7 (43.75)		
Total thyroidectomy			0.001	0.978
Yes	136 (87.74)	14 (87.50)		
No	19 (12.26)	2 (1.25)		
Laryngeal or trachea invasion			1.261	0.262
Yes	30 (19.35)	5 (31.25)		
No	125 (80.65)	11 (68.75)		
Esophagus invasion			5.288	0.021
Yes	23 (14.84)	6 (3.75)		
No	132 (85.16)	10 (6.25)		
RLN invasion location			0.689	0.406
Recurrent laryngeal nerve inlet zone	21 (13.55)	1 (6.25)		
Others	134 (86.45)	15 (93.75)		
RLN condition			0.773	0.379
Invasion	79 (50.97)	10 (6.25)		
Adhesion	76 (49.03)	6 (3.75)		
RLN invasion condition			1.116	0.291
Primary tumor	123 (79.35)	15 (93.75)		
LNM	32 (20.65)	1 (6.25)		
Surgical methods of RLN			0.944	0.331
Resection	87 (56.13)	11 (68.75)		
Separation or PLR	68 (43.87)	5 (31.25)		
CLNM ≥ 5			0.075	0.784
Yes	29 (18.71)	4 (25.00)		
No	126 (81.29)	12 (75.00)		
LLNM			0.635	0.425
Yes	71 (45.81)	9 (56.25)		
No	84 (54.19)	7 (43.75)		
Hashimoto’s thyroiditis			0.000	1.000
Yes	15 (9.68)	2 (12.50)		
No	140 (90.32)	14 (87.50)		
Postoperative RAI therapy			0.180	0.672
Yes	113 (72.90)	13 (81.25)		
No	42 (27.10)	3 (18.75)		

PTC, papillary thyroid carcinoma; VCP, vocal cord paralysis; RLN, recurrent laryngeal nerve; PLR, partial layer resection; CLNM, central lymph node metastasis; LLNM, lateral lymph node metastasis; RAI, radioactive iodine.

### Univariable and multivariable analyses of the risk of recurrence

As shown in **Table 5**, among the various clinicopathological characteristics, age ≥ 55 years, preoperative VCP, and esophageal invasion were associated with an increased risk of PTC recurrence in univariate analyses (P < 0.100). However, only age ≥ 55 years was confirmed in the multivariable analysis (hazard ratio 4.52, 95% confidence interval: 1.44–14.19, P = 0.010).

**Table 5:** Univariable and multivariable Cox proportional hazards regression analyses of the risk of recurrence.

Characteristics	Univariate analysis		Multivariate analysis	
	Hazard ratio (95% CI)	*P*	Hazard ratio (95% CI)	*P*
Sex				
Female				
Male	1.23 (0.45–3.40)	0.685		
Age ≥ 55 years				
No				
Yes	4.99 (1.61–15.50)	**0.005**	4.52 (1.44–14.19)	0.010
BMI (kg/m^2)^	0.98 (0.84–1.14)	0.782		
Tumor size (cm)	1.10 (0.90–1.35)	0.342		
Multifocal disease				
No				
Yes	1.02 (0.38–2.72)	0.970		
VCP				
No				
Yes	2.37 (0.88–6.37)	0.088	1.66 (0.59–4.65)	0.334
Total thyroidectomy				
Yes				
No	0.83 (0.19–3.65)	0.804		
Laryngeal or trachea invasion				
No				
Yes	1.96 (0.67–5.68)	0.217		
Esophagus invasion				
No				
Yes	3.12 (1.13–8.59)	**0.028**	2.44 (0.85–6.99)	0.096
RLN condition				
Adhesion				
Invasion	1.62 (0.59–4.47)	0.349		
RLN invasion condition				
Primary Tumor				
LNM	0.30 (0.04–2.28)	0.245		
Surgical methods of RLN				
Separation or				
Resection	1.64 (0.57–4.73)	0.357		
Lymph node metastasis				
Yes				
No	1.06 (0.37–3.05)	0.918		
CLNM ≥ 5				
Yes				
No	0.62 (0.20–1.91)	0.401		
LLNM				
Yes				
No	0.59 (0.22–1.59)	0.298		
Hashimoto’s thyroiditis				
No				
Yes	1.48 (0.34–6.53)	0.603		
Postoperative RAI therapy				
Yes				
No	0.53 (0.15–1.86)	0.322		

BMI, body mass index; PTC, papillary thyroid carcinoma; VCP, vocal cord paralysis; RLN, recurrent laryngeal nerve; PLR, partial layer resection; CLNM, central lymph node metastasis; LLNM, lateral lymph node metastasis; RAI, radioactive iodine.

**Figure 3:**
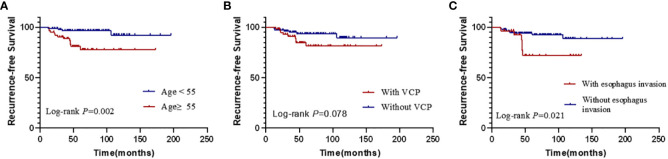
Kaplan–Meier curves for the recurrence-free survival according to different risk factors of univariable analysis. **(A)** the differences of RFS according to age **(B)** the differences of RFS according to the preoperative condition of the vocal cords. **(C)** the differences of RFS according to the status of esophagus. (VCP, vocal cord paralysis).

**Figure 4:**
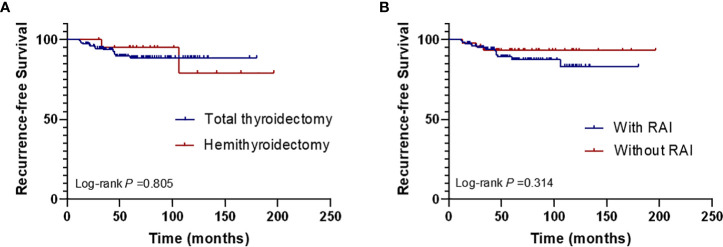
Kaplan–Meier curves for the recurrence-free survival according to the surgical scope of thyroid **(A)** and the postoperative RAI therapy **(B)**. (RAI, radioactive iodine).

Kaplan–Meier analysis showed that the RFS rate was significantly lower in patients aged ≥ 55 years than in those aged < 55 years (*P* = 0.002). Patients with esophageal invasion also had significantly lower RFS (*P* = 0.021). In addition, patients with preoperative VCP seemed to have a worse RFS than those without VCP, although this did not reach statistical significance (*P* = 0.078) (**Figure 3**). Other clinical characteristics were also suspected to be related to recurrence, such as the surgical scope and postoperative RAI therapy. To figure out their relationship with recurrence, we also performed Kaplan–Meier analysis. The results showed that there were no differences between patients who underwent total thyroidectomy and those who had a hemithyroidectomy (*P*=0.805) or patients who received or did not receive postoperative RAI (*P*=0.314) (**Figure 4**).

## Discussion

PTC is the most common type of malignant endocrine tumor. Although the majority are generally well-differentiated and demonstrate a favorable outcome, PTC is highly invasive in some patients, with the tumor directly invading into the surrounding tissue and structures. The RLN is usually located in the tracheoesophageal groove, and when the thyroid tumor penetrates the latter thyroid capsule or when the LNs of this region are metastatic, the RLN may become involved, and the function of the nerve may be impacted. Research on these patients’ survival outcomes and risk factors for recurrence can provide a scientific basis for the precise management of patients with PTC.

Assessment of RLN invasion includes preoperative and intraoperative observations. Because the RLN is typically located deep in the neck, it is difficult to estimate the condition of the RLN using routine imaging examinations. Some studies have reported that the preoperative assessment of RLN condition using CT, MRI or US. These imaging examinations may have given surgeons hints about the possibility of RLN invasion, however the sensitivity and accuracy were not satisfactory (9–11). Laryngoscopic examination is routinely recommended, but some patients with RLN invasion are still asymptomatic. In this study, only 60 patients experienced preoperative VCP and were identified using laryngoscopy. Other studies have reported that approximately 50–60% of invaded RLNs presented with normal preoperative nerve function (12, 13). Alexandre Dahan et al. reported that even in PTC patients whose RLNs were encased by thyroid tumors, preoperative VCP was present in 16/52 cases (30%) (14). It is more difficult to identify patients with RLN invasion without preoperative VCP. Since the preoperative assessment of RLN condition is often difficult, numerous guidelines now focus more on intraoperative RLN assessment, suggesting that intraoperative neural monitoring (IONM) is necessary for the optimal management of RLN invaded by thyroid tumor (3, 7, 15). As voice analysis continues to be refined and improved, it may provide new ideas and approaches for assessing the RLN preoperatively and noninvasively. Voice analysis is often used to evaluate voice quality in diverse dimensions. Several studies have performed voice analysis in PTC patients postoperatively and found that surgery has side effects on voice quality (16–18). To the best of our knowledge, no previous study had investigated the association between RLN condition and preoperative voice analysis findings. We found that in cases with RLN invasion, jitter and shimmer increased significantly, while MPT decreased significantly, indicating that the stability of the vocal cords worsened. Similarly, in patients with VCP, the jitter and shimmer increased significantly, and the MPT decreased significantly. These findings indicate that preoperative voice analysis can provide efficient and effective information regarding RLN conditions.

The association between age and the prognosis of thyroid cancer has long been a concern. In a previous study, Shah et al. reported that age was a key predictor of response to therapy and disease-specific survival in ATA high-risk thyroid cancer patients (19). Other studies have also indicated that age plays an important role in the survival of patients with ATA high-risk thyroid cancer (20–22). These findings indicated that it was appropriate to use age 55 years as the cut-off when considering tumor stage.

According to the AJCC Cancer Staging Manual, 8^th^ edition, PTC patients with RLN invasion are assigned to stage T4a. In many cases, patients often suffer not only invasion of the RLN, but also of other structures, such as the trachea, larynx, and esophagus. There is still some debate regarding the influence of RLN invasion on survival. Indeed, several retrospective studies have found that PTC patients with only RLN invasion seem to have a better prognosis than patients with other extrathyroidal invasions (23, 24). Thus, some researchers have called for downstaging of patients with invasion of only the RLN. However, some previous studies have also shown that even though there was only RLN invasion, the OS and RFS rates were still lower than those in patients without any extrathyroidal invasion (25–27). In this study, we found that patients with esophageal invasion had significantly lower RFS than those without esophageal invasion, and that the RFS of patients with preoperative VCP tended to be lower than that of patients without preoperative VCP, although that difference was not statistically significant. These findings emphasize the need to actively manage these patients. The actual role of RLN invasion in the survival prognosis of patients requires further in-depth studies.

This study was limited in that it was a retrospective, single-center study. The long-term voice data were missing, making it hard to compare the voice quality between patients receiving RLN reconstruction or not. It was also subject to the inherent limitations associated with retrospective analyses. Multi-institutional studies with larger sample sizes are needed to confirm the results of our study.

## Conclusion

Patients with PTC with RLN invasion are commonly encountered in clinical practice. To achieve optimal survival and vocal outcomes, comprehensive preoperative examinations are necessary. Our study provided novel insights into the use of voice analysis to evaluate RLN function. Moreover, survival analysis demonstrated that age of onset greater ≥ 55 years was an independent risk factor for postoperative recurrence in T4a PTC patients.

## Data availability statement

The original contributions presented in the study are included in the article/supplementary material. Further inquiries can be directed to the corresponding authors.

## Author contributions

QZ: review and editing (equal). LH, HM, QS: Conceptualization (lead); writing – original draft (lead); formal analysis (lead); writing – review and editing (equal). LF, XS, RW: Software (lead); writing – review and editing (equal). SH, YL, ML: Methodology (lead); writing – review and editing (equal). JF, JC: Conceptualization (supporting); Writing – original draft (supporting); Writing – review and editing (equal). All authors contributed to the article and approved the submitted version.

## Funding

This study was supported by the National Key R&D Program of China (2020YFB1312805), Capital Health Research and Development of Special (2022–1–2051), Beijing Municipal Administration of Hospitals’ Youth Programme (QMS20210206); Scientific Research Common Program of Beijing Municipal Commission of Education (KM202210025014), Beijing Municipal Administration of Hospitals’ Ascent Plan (DFL20180202), Scientific Research and Incubating Fund of Capital Medical University(PYZ21096).

## Conflict of interest

The authors declare that the research was conducted in the absence of any commercial or financial relationships that could be construed as a potential conflict of interest.

## Publisher’s note

All claims expressed in this article are solely those of the authors and do not necessarily represent those of their affiliated organizations, or those of the publisher, the editors and the reviewers. Any product that may be evaluated in this article, or claim that may be made by its manufacturer, is not guaranteed or endorsed by the publisher.
